# Highly Filled Waste Polyester Fiber/Low-Density Polyethylene Composites with a Better Fiber Length Retention Fabricated by a Two-Rotor Continuous Mixer

**DOI:** 10.3390/polym16202929

**Published:** 2024-10-18

**Authors:** Junrong Chen, Zhijie Pan, Songwei Yang, Changlin Cao, Weiming Zhou, Yidu Xie, Yilin Yang, Qingrong Qian, Qinghua Chen

**Affiliations:** 1 Engineering Research Center of Polymer Green Recycling of Ministry of Education, Key Laboratory of Pollution Control & Resource Reuse, College of Environmental and Resource, College of Carbon Neutral Modern Industry, Fujian Normal University, Fuzhou 350007, China; chjrong04@163.com (J.C.); panzj0815@163.com (Z.P.); wmzhou@fjnu.edu.cn (W.Z.); xieyidu@126.com (Y.X.); qrqian@fjnu.edu.cn (Q.Q.); cqhuar@fjnu.edu.cn (Q.C.); 2 Quangang Petrochemical Research Institute, Fujian Normal University, Quanzhou 362807, China; 3 Fujian Nan’an Shida Rubber & Plastic Machinery Co., Ltd., Quanzhou 362314, China; yangyilin1970@126.com

**Keywords:** waste polyester fiber, twin-rotor continuous mixer, high fiber content, fiber length

## Abstract

A key challenge in the utilization of waste polyester fibers (PET fibers) is the development of fiber-reinforced composites with high filler content and the improvement of fiber length retention. Herein, the effects of a two-rotor continuous mixer and a twin-screw extruder on the structure and properties of waste polyester fiber composites were evaluated. The results revealed that the mechanical properties of the composites were improved significantly with increasing fiber content, especially when processed using the twin-rotor continuous mixer. This mixer facilitated the formation of a robust fiber network structure, leading to substantial enhancements in tensile strength, flexural strength, and heat resistance. Specifically, compared to those processed by the twin-screw extruder, with 60 wt% fibers content, the tensile and flexural strengths of specimens processed by the twin-rotor continuous mixer increase by 21% and 13%, respectively. The average fiber length in specimens processed by the twin-rotor continuous mixer was 32% longer than that in specimens processed by the twin-screw extruder, attributable to the lower shear frequency and the higher tensile ratio of the former. This blending technique emerges as an effective strategy, contributing significantly to promoting the development and practical application of waste textile fiber-reinforced polymer composites.

## 1. Introduction

Textiles, integral to human life, span various applications including daily clothing, household goods, and packaging materials, and form a crucial component of modern material culture. Their production and consumption have experienced substantial growth, with polyester fibers holding a dominant position in the textile market [1,2]. However, this rapid expansion of the textile industry is accompanied by significant environmental and resource challenges, particularly concerning the disposal of textile waste, which has become a critical global environmental issue [3,4].

Utilizing polyester-based waste textile fibers as fillers in polyolefins is highly significant for pollution reduction, carbon reduction, and advancing a circular economy [5,6]. This approach can mitigate the environmental impact of waste textiles, converting low-value waste polyester fibers into high-performance, value-added fiber-reinforced materials. This not only reduces production costs but also enhances resource recycling and market competitiveness, thus providing new opportunities for industry development [7,8].

Polyester fibers are polar, while polyolefins are non-polar, and the poor compatibility between the two materials leads to poor performance of the fiber-reinforced composites, so the amount of fibers added is limited [9]. Increased fiber filling exacerbates issues such as stress concentration from fiber agglomeration, and inadequate dispersion [10,11,12,13]. Wu et al. demonstrated that the properties of fiber-reinforced composites can be improved by adding maleic anhydride-grafted polyolefins to increase compatibility [14]. These polar groups can form chemical bonds or strong physical adsorption with the fiber surface, thereby improving material properties [15].

The reinforcement mechanism in fiber-reinforced materials relies on the high strength and stiffness of the fibers, which transfer stress between the matrix and fibers during interfacial shear [16,17]. Thus, composite strength depends not only on the properties of the fibers and the matrix material but also on the fiber length. Traditional methods for mixing and dispersing fibers with resin, such as using compactors and screw extruders, face challenges in maintaining fiber length and accommodating high filler systems [18]. Recently, the twin-rotor continuous mixer, which offers superior dispersion and mixing capabilities combined with continuous processing advantage, has garnered significant attention. Yu et al. demonstrated that a twin-rotor continuous mixer better preserved glass fiber length in reinforced polypropylene composites compared to a twin-screw extruder [19]. This indicates the potential for using a twin-rotor continuous mixer in producing waste textile fiber-reinforced polyolefin composites, though current research primarily focuses on low fiber content, highlighting the need for further exploration of high fiber content applications.

In this study, recycled waste polyester fiber served as filler material, maleic anhydride grafted low-density polyethylene (LDPE) was employed as the compatibilizer, and LDPE functioned as the matrix. The investigation systematically assessed the structure and properties of waste polyester fiber-reinforced composite materials prepared using both a twin-screw extruder and a twin-rotor continuous mixer, focusing on various fiber filler amounts. This research provides insights for the development of waste fiber composites with high fiber content.

## 2. Materials and Methods

### 2.1. Materials

The waste polyester fiber (PET fiber) was collected from scraps by Huafeng Huajin Co., Ltd. (Putian, China) during the production process. Xylene, an analytical reagent, was purchased from Sinopharm Chemical Reagent Co., Ltd. (Shanghai, China). LDPE was purchased from BASF-YPC Co., Ltd. (Nanjing, China). Low-density polyethylene grafted with maleic anhydride (LDPE-g-MAH, W1L-1) was purchased from COACE Chemical Co., Ltd. (Xiamen, China).

### 2.2. Preparation Method

#### 2.2.1. Pre-Coating of Fibers

The LDPE, LDPE-g-MAH, and waste polyester fibers were dried in an oven at 100 °C for 8 h. According to the formula in Table 1, LDPE-g-MAH and waste PET fibers were put into a two-roll open mill (ZG-80T, Zhenggong Electromechanical Equipment Technology Co., Ltd., Dongguan, China) at a temperature of 120 °C. LDPE-g-MAH was gradually melted and wrapped around the fibers through the shear of two rollers. Subsequently, LDPE was added to the fibers for wrapping. The pre-blend with wrapped fibers was then fed into a crusher and ground into particles smaller than 5 mm. The pre-coating process allowed the loose fibers and LDPE into the mixing chamber at the same time without being bounced up by the screw, ensuring a uniform feed.

#### 2.2.2. Blending and Extrusion of Waste PET Fiber/LDPE Composites

Through a twin-screw extruder (MEDI-22/40, Guangzhou Putong Experimental Analysis Instrument Co., Ltd., Guangzhou, China) and twin-rotor continuous mixer (CM-35, Fujian Nan’an Shida Rubber & Plastic Machinery Co., Ltd., Quanzhou, China), the pre-blended material was blended, extruded, and injected into the sample for the mechanical properties test through the injection molding machine.

“SPn” and “RPn” designations were used for the samples. “SP” denoted those samples prepared by the twin-screw extruder, “RP” denoted those samples prepared by the twin-rotor continuous mixer, and “n” denoted the added amount of waste fiber.

### 2.3. Characterization

#### 2.3.1. Length Distribution of Fibers

**Figure 1:**
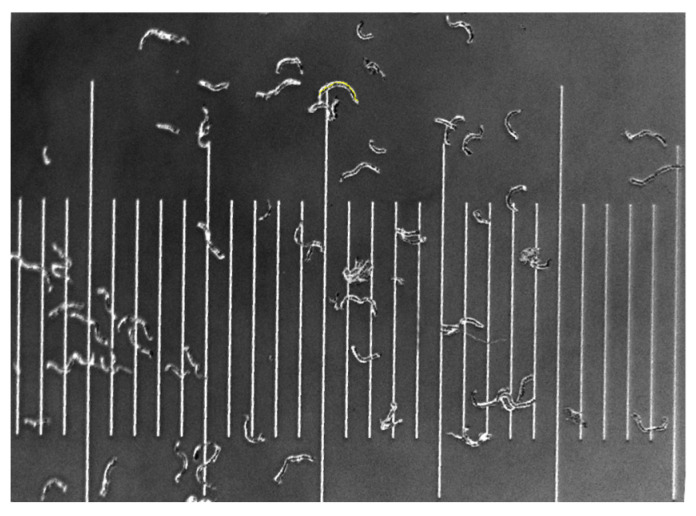
Measurement of the length map using ImageJ software, and a small grid of the scale is 0.1 mm.

The fibers in S60 and R60 were extracted using the soxhlet extraction method and xylene as a solvent. The samples (5 g) were accurately weighed and placed in a copper net, after extracting at 180 °C for 24 h [20], then dried at 80 °C in a vacuum drying chamber to constant weight for 12 h. The fibers were laid flat on the conductive adhesive, the laminated fibers were removed and photographed with an optical microscope. The fiber length was measured and counted by ImageJ software (version 1.54k) (as shown in Figure 1), with 200 fibers counted for each sample. As shown in Figure 1, a small grid of the scale is 0.1 mm. The length of the fibers was calculated by ImageJ software after scanning the contours of the fibers with reference to the scale. The arithmetic mean was calculated by statistical software (Origin 2021).

#### 2.3.2. Mechanical Properties Test

The tensile properties of the samples were tested in accordance with GB/T 1040 [21] by a universal testing machine (CMT4104, Nss Laboratory Equipment Co., Ltd., Shenzhen, China) at a speed of 50 mm/min. The flexural properties of the samples were tested in accordance with GB/T 9341 [22] by a universal testing machine. The impact strengths of the samples were tested in accordance with GB/T 1843 [23] by a universal testing machine.

#### 2.3.3. Scanning Electron Microscopy (SEM)

Microscopic morphology of sample sections after the impact strength testing was obtained from a scanning electron microscope (SEM, SU8100, Hitachi High-Tech, Tokyo, Japan). Before the test, the samples were coated with a layer of Au.

#### 2.3.4. Differential Scanning Calorimeter (DSC)

The glass transition temperature was measured by a differential scanning calorimeter (DSC, Q20, TA Instruments, New Castle, PA, USA). The samples were weighed 5~10 mg and placed in aluminum crucible. In a nitrogen atmosphere, the sample was heated from 30 °C to 280 °C at a rate of 10 °C. The relative crystallinity was calculated by Equation (1) [24].

(1) $$\chi_{c}=\frac{\Delta H_{m}}{\Delta H_{m}^{0}\times \omega }\cdot 100\%$$

where $\Delta H_{m}$ denotes the enthalpy of melting, J·g^−1^; $\Delta H_{m}^{0}$ is the enthalpy of melting of LDPE and PET when they are fully crystallized and is taken as 293 J·g^−1^ for LDPE and 140 J·g^−1^ for PET; and ω is the mass fraction of LDPE and PET.

#### 2.3.5. Dynamic Mechanical Analysis (DMA)

A dynamic mechanical analyzer (DMA, Q800, TA Instruments, New Castle, PA, USA) was utilized to test the dynamic thermomechanical properties of the samples. The length, width, and thickness of the test specimen were 60 × 10 × 4 mm. The test mode of temperature scanning was used, and the test fixture was a double cantilever fixture. The fixed test amplitude was 10 μm, the frequency was 1 Hz, and the test temperature was increased from 30 °C to 100 °C at 3 °C/min.

#### 2.3.6. Dynamic Rheological Analysis (DRA)

A rotational rheometer (DRA, DHR-2, TA Instruments, New Castle, PA, USA) was used to test the rheological behavior of the samples. An aluminum parallel plate fixture with a diameter of 8 mm was used, and the test spacing and temperature were 1 mm and 190 °C, respectively. The strain was 0.1%, and the angular frequency scanning range was 0.1–100 rad/s. The test was carried out using a rotational rheometer.

## 3. Results and Discussion

### 3.1. Analysis of the Length Distribution of Fibers

Equation (4) can be obtained by combining Equation (2) for the critical fiber length in the Kelly–Tyson model and Equation (3) for the strength of the fiber composite [25,26,27]. It can be seen that for the same fiber content, the fiber length in the composite determines the final strength of the composite.

(2) $$L_{c}=\frac{\sigma_{f}\cdot d}{2\tau }$$

(3) $$\sigma_{c}=\sigma_{m}(1-V_{f})+\sigma_{f}V_{f}\frac{L}{L_{c}}$$

(4) $$\sigma_{c}=\sigma_{m}(1-V_{f})+V_{f}\frac{L}{d}2\tau$$

where L_c_ is the critical fiber length, σ_f_ is the tensile strength of the fibers, d is the diameter of the fibers, τ is the interfacial shear strength, σ_c_ is the strength of the composite, σ_m_ is the strength of the matrix material, V_f_ is the volume fraction of the fibers, and L is the actual length of the fibers.

**Figure 2:**
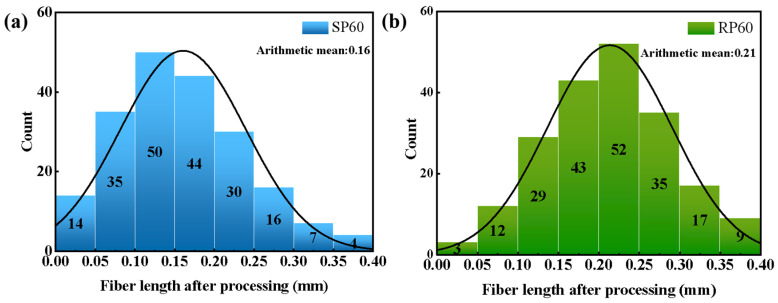
Histogram of SP60 and RP60 fiber length distribution after processing, (**a**) SP60 and (**b**) RP60.

It was seen from the equation that the length of the processed fiber determined the ability of the composite to withstand loads when the fiber type and content were the same, and it was essential to maintain the length of the fibers during processing. The results of counting the fiber length distribution in SP60 and RP60 are shown in Figure 2. Calculating the arithmetic mean of fiber lengths, it could be seen that SP60 had an average length of 0.16 mm, while RP60 had an average length of 0.21 mm. SP60 demonstrated a higher distribution frequency of shorter fiber lengths compared to RP60, indicating that the two-rotor continuous mixer was more effective at preserving fiber length during the mixing process.

**Figure 3:**
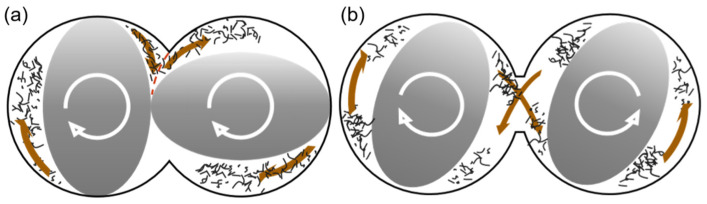
Schematic cross-section of the mixing chamber of the twin-screw extruder and the two-rotor continuous mixer: (**a**) twin-screw extruder and (**b**) two-rotor continuous mixer.

The superior performance of the twin-rotor continuous mixer compared to the twin-screw extruder in maintaining processed fiber length is attributed to the significant differences in the mixing mechanisms of the two machines and the subjected shear frequency of fibers during the mixing process. Figure 3 shows a schematic diagram of the cross-section of the mixing chamber of the twin-screw extruder and the twin-rotor continuous mixer. As depicted in Figure 3a, during the mixing process of the twin-screw extruder, the space between the front space in the direction of rotation of the screw prong and the other screw prong gradually diminishes, subjecting the fibers to a strong shear effect that disperses them between the left and right chambers, resulting in a higher shear frequency. In contrast, Figure 3b shows that in the mixing process of the two-rotor continuous mixer, fibers are exchanged between the two rotors. The shear effect primarily occurs during the extrusion and stretching as the screw-pronged rotor slides along the wall of the mixing chamber, resulting in fibers being subjected to a lower shear frequency.

### 3.2. Mechanical Properties Analysis

**Figure 4:**
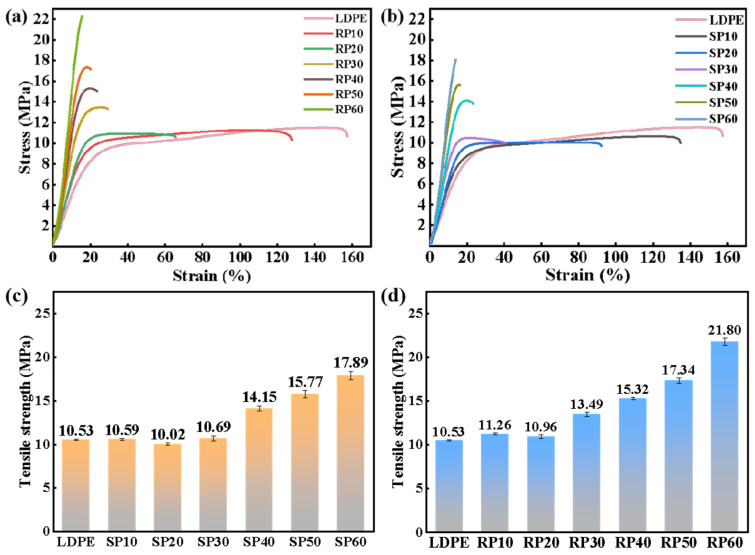
Mechanical properties of SP and RP composites with different fiber contents: (**a**,**b**) stress–strain curves and (**c**,**d**) tensile strength.

**Figure 5:**
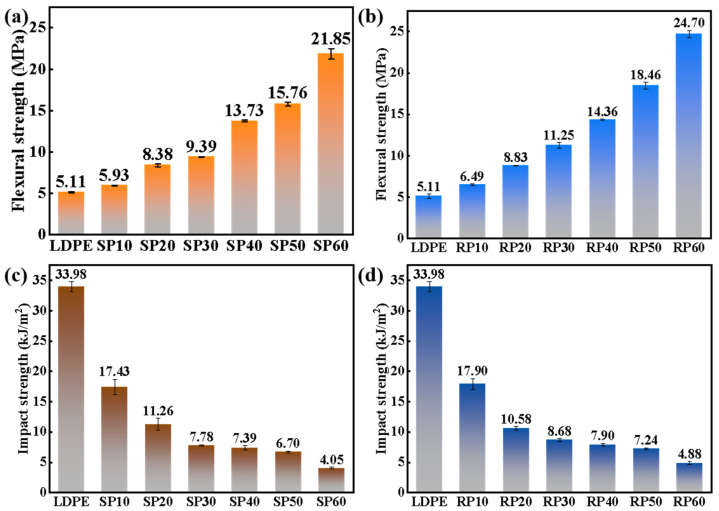
Mechanical properties of SP and RP composites with different fiber contents: (**a**,**b**) flexural strength and (**c**,**d**) impact strength.

Figure 4 and Figure 5 show the mechanical properties of SP and RP samples with different fiber contents. As observed in Figure 4c,d, the tensile properties of both SP and RP improved with the increase in fiber content. This enhancement occurred because the force was transferred to the fibers through the interfacial shear between LDPE and fibers when the material was subjected to tensile force [28]. The fibers had a higher strength than LDPE to withstand a larger load, thereby improving the tensile properties of the composites. When the fiber content was increased to 60%, the tensile properties of SP60 and RP60 improved by 69% and 107%, respectively, compared to LDPE. This indicates that the fibers had a significant reinforcing effect on LDPE. The elongation at the break of the samples decreased significantly with the increase in fiber content, as shown in Figure 4a,b, exhibiting a significant brittle behavior. This was attributed to the stress concentration caused by fiber debonding or breaking under tensile loading [29], which increased the rate of crack extension and led to a reduction in elongation at break.

Comparing the trends in tensile properties of SP and RP, it was found that a significant increase in tensile properties occurred for SP when the fiber content exceeded 30%, while for RP, a significant increase was observed when the fiber content exceeded 20%.

As shown in Figure 5a,c, the flexural strength of both SP and RP increased with the addition of fiber content, indicating a significant improvement in the rigidity of the samples. This was attributed to the incorporation of rigid polyester fibers, which improve the sample’s rigidity. Furthermore, it was observed that the flexural strength of RP was generally better than that of SP. This may be attributed to the longer fibers confining the LDPE chain segments, reducing slippage and thereby increasing the rigidity of RP when subjected to flexural forces.

From Figure 5c,d, it can be observed that the impact properties of SP and RP gradually decreased with the increase in fiber content, indicating a reduction in the toughness of the samples. This was due to the higher fiber content hindering the molecular chain movement of polyethylene, leading the composite material to adopt a more rigid segment structure and resulting in increased brittleness.

### 3.3. Micromorphology Analysis

**Figure 6:**
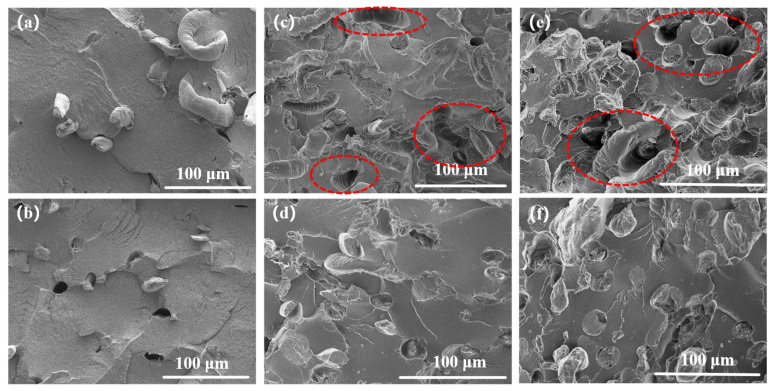
Section morphology of SP and RP composites with different fiber contents: (**a**) SP10; (**b**) SP30; (**c**) SP50; (**d**) RP10; (**e**) R350; (**f**) RP50.

Figure 6 shows the section morphology of SP and RP with different fiber contents. The pits and protrusions resulting from fiber pullout and debonding are clearly visible in Figure 6a–c. The twin-screw extruder continuously shears and converges the material at the engaging screw prongs, causing the fibers to be repeatedly compressed and entangled. Consequently, the fibers in SP can be seen in the figure with more obvious agglomerations in the section. In contrast, the twin-rotor continuous mixer helped to straighten and untwist fibers and reduced the chance of fiber agglomeration through the coiling action between the rotor prongs. The material was pushed and pulled in the direction of extrusion, with the material between the prongs and the mixing chamber wall being squeezing, rubbing, and stretching. The reduced fiber agglomeration and improved fiber orientation enhance the efficiency of load transfer by the fibers, leading to greater load-bearing capacity [30].

### 3.4. Analysis of DSC

**Figure 7:**
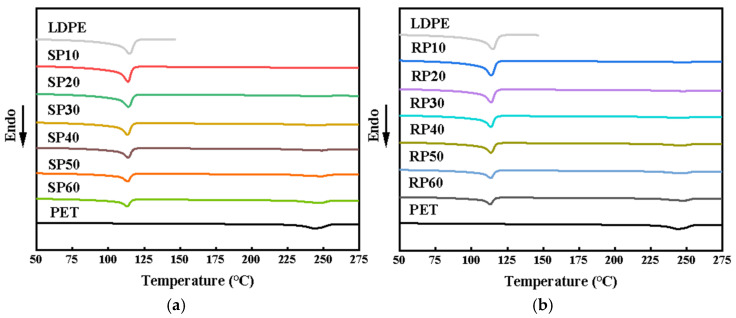
DSC rising temperature curves of SP and RP composites with different fiber content: (**a**) SP composites and (**b**) RP composites.

Figure 7 shows the DSC curves of SP and RP with different fiber contents. The corresponding thermal property data are displayed in Table 2, which include the melting temperature (T_m_), enthalpy of melting (ΔH_m_), and crystallinity (χ_c_). It is observed that both SP and RP exhibit melt peaks for LDPE and PET, with similar T_m_ between the components, indicating no structural changes at the interface [29]. An increase in fiber content impedes the movement of LDPE molecular segments, leading to a decrease in T_m_ [31]. As shown in Table 2, T_m_ (PE) slightly decreases with increasing fiber content; however, this change is likely due to instrumental uncertainty as it too minor to be significant. The ΔH_m_ and χ_c_ of the representative fibers exhibit a clear trend. The χ_c_ of the fibers in SP decreases with increasing fiber content and is consistently lower than that of RP at the same fiber content. Since the processing temperature is always below the melting point of the fibers, the molecular chain segments do not undergo drastic movement during processing. This change in crystallinity is attributed to the fibers being cut off. The shearing action during processing disrupts the original arrangement of the molecular chain segments, preventing them from extending and connecting to form a complete crystal structure.

### 3.5. Dynamic Mechanical Analysis

**Figure 8:**
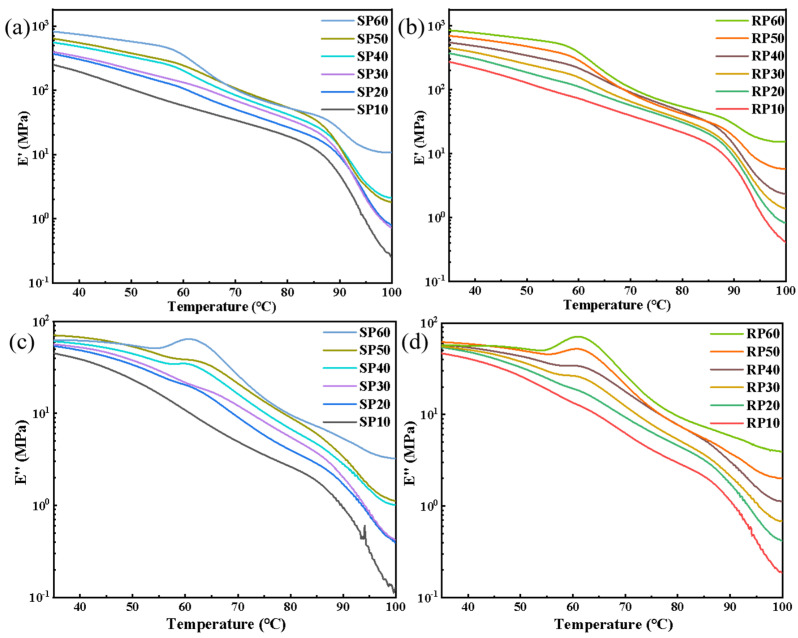
DMA curves of SP and RP composites with different fiber contents: (**a**,**b**) storage modulus; (**c**,**d**) loss modulus.

Figure 8a,b show the energy storage modulus (E’) curves for SP and RP with different fiber contents. The E’ decreased with increasing temperature and increased with increasing fiber content. This trend is attributed to the stress transfer from LDPE to the fibers, where the rigid fibers act as a reinforcing agent. The presence of fibers restricts the movement of LDPE chain segments, requiring more energy to overcome the intermolecular forces, thereby increasing the E’ of the system [32]. At the same temperature, the E’ of RP was generally higher than that of SP, indicating the superior heat resistance and broader service temperature of RP.

Figure 8c,d show the loss modulus (E″) curves for SP and RP with different fiber contents. There is a general decreasing trend in the E″ with increasing temperature, particularly at low fiber content (e.g., 10%). The curves exhibit a distinct peak when the fiber content is increased to 30% for RP and 40% for SP. This peak is due to the fact that, with increasing temperature, the molecular chain segments begin short-range movement and rearrangement [33]. The LDPE undergoes thermal deformation and gradually softens, but the presence of internal fibers limits the movement of the LDPE chain segments, increasing the internal friction between them.

### 3.6. Dynamic Rheological Analysis

**Figure 9:**
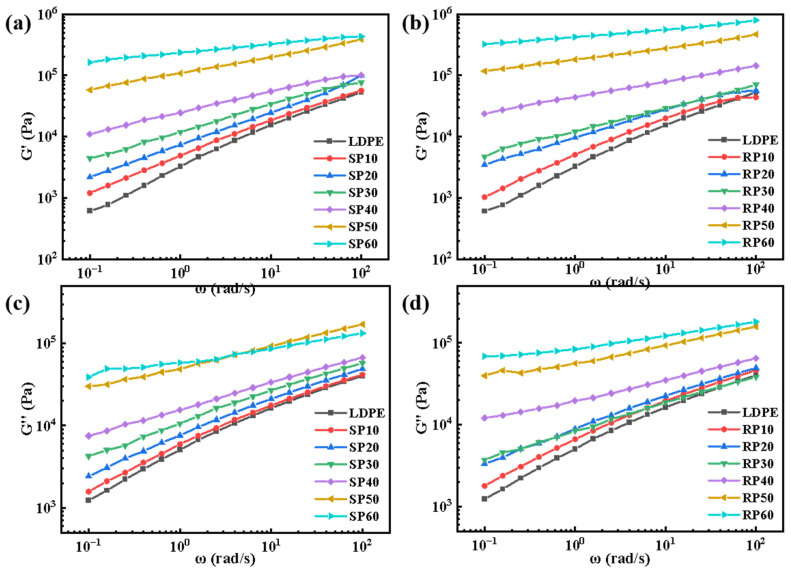
Dynamic frequency sweep curves of SP and RP composites with different fiber contents: (**a**,**b**) storage modulus; (**c**,**d**) loss modulus.

Figure 9 shows the curves of the energy storage modulus (G’) and loss modulus (G″) versus angular frequency for SP and RP with different fiber contents. Both the G’ and G″ increased with frequency, peaking at a 60% fiber addition, which demonstrates the reinforcing effect of fibers on LDPE. For the same fiber content, the G’ and G″ of RP are higher than those of SP, indicating that RP possesses better stiffness.

In the case of SP, when the fiber content was 10%, 20%, and 30%, both the G’ and G″ of the material increased with angular frequency (ω), exhibiting linear characteristics [34]. When the fiber content was 40%, 50%, and 60%, the G’ and G″ plateaued in the low-frequency range, showing nonlinear characteristics. This suggests that the material responded more slowly in the low-frequency region, requiring more time for energy transfer and deformation. The increase in fiber content further impeded the movement of LDPE chain segments, leading to longer rearrangement times for the molecular chains. An energy dissipation phenomenon occurred in the low-frequency region, intensifying with increasing fiber content, which resulted in more pronounced energy loss and the formation of an energy storage modulus plateau. RP formed a plateau in the low-frequency region with fewer fibers added than SP. This was attributed to the higher fiber retention in RP, where longer fibers increased the potential for entanglement, making energy dissipation more apparent.

**Figure 10:**
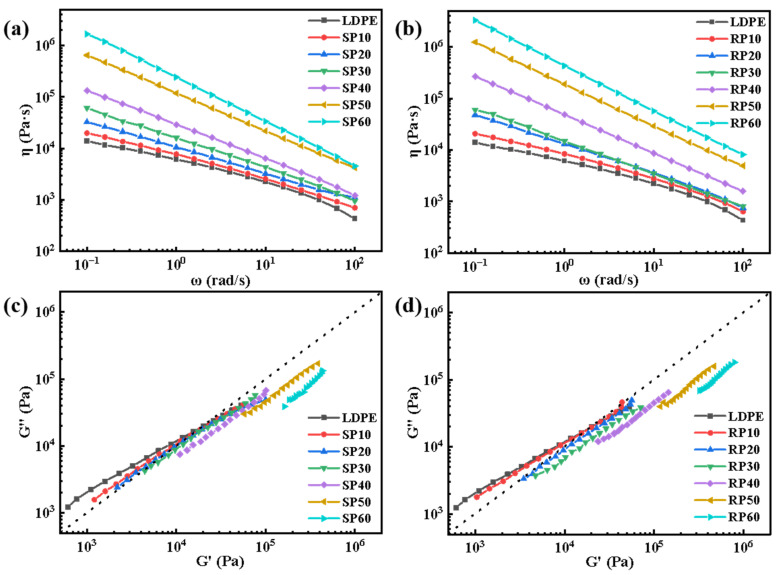
Dynamic frequency sweep curves of SP and RP composites with different fiber contents: (**a**,**b**) complex viscosities; (**c**,**d**) Han diagram.

Figure 10 shows the complex viscosity versus angular frequency curves along with the Han diagram for SP and RP with different fiber contents. The viscosities of both SP and RP increased with fiber content and exhibited non-Newtonian characteristics across all frequency ranges [35]. This increase in viscosity is attributed to the presence of fibers that impeded the normal flow of LDPE chain segments. Both SP and RP exhibited typical shear thinning behavior throughout the frequency range. This behavior is due to the structural rearrangement of fibers and LDPE: as the frequency of the applied shear stress increases, the viscosity of the composites decreases. Similar to the elastic modulus plots, the viscosity curves show a more pronounced plateau at fiber contents of 10% and 20%. This plateau is due to the structural rearrangement of the disordered distribution of fibers dominating the flow behavior and slowing down the shear thinning effects at lower fiber contents. The diagonal line in the Han curve graph represents the equal modulus line, which is commonly used to illustrate shifts in the viscoelastic properties of the material [36]. As fiber content increased, the Han curve of the material gradually shifted to the lower right. This shift indicates that the addition of fibers drives the composite towards a solid-like state, with the fibers forming a three-dimensional network structure within the material.

## 4. Conclusions

In this study, waste PET fiber/LDPE composites were fabricated using a two-rotor continuous mixer and compared with the samples prepared by a twin-screw extruder. The results indicate that an increase in fiber content significantly improved the tensile and flexural strengths of the waste fiber composite materials but reduced the impact strength. The reduction in impact strength is associated with the decreased continuity of LDPE due to an excess of fibers, which also led to the concentration of stress at fiber aggregation points. Compared to the twin-screw extruder, the two-rotor continuous mixer processing had a lower fiber shear frequency and a higher stretch ratio, which better maintained fiber length and improved the mechanical properties of the composites, particularly at high fiber additions. Furthermore, with the increase in PET fiber content, the heat resistance of the composites was improved. While a 30% fiber content in SP was needed for the sample’s structure to transition from liquid-like to solid-like, RP achieved this effect at 20% fiber content. It was observed that the two-rotor continuous mixer processing of the fiber-reinforced materials could more efficiently utilize the physical properties of fibers, thereby enhancing the properties of composites. These findings suggest that the two-rotor continuous mixer can better retain fiber length, leading to improved properties of fiber-reinforced composite materials. Given the substantial industry demand in the future and societal policies of non-pollution, green, and low-carbon practices. The two-rotor continuous mixer, with its shear and tensile flow fields, is an ideal choice for advancing the fiber recycling industry and the industrial upgrading of fiber-reinforced composite materials.

## Figures and Tables

**Table 1:** Formulation of waste fiber/LDPE composites.

Sample	Waste PET Fiber (wt%)	LDPE (wt%)	LDPE-g-MAH (wt%)
LDPE	0	100	0
SP10/RP10	10	89	1
SP20/RP20	20	78	2
SP30/RP30	30	67	3
SP40/RP40	40	56	4
SP50/RP50	50	45	5
SP60/RP60	60	34	6

**Table 2:** Thermal properties of SP and RP composites with different fiber contents.

	T_m_ (PE)/°C	∆H_m_ (PE)/J·g^−1^	χ_c_ (PE)/%	T_m_ (PET)/°C	∆H_m_ (PET)/J·g^−1^	χ_c_ (PET)/%
LDPE	114.8	138.2	47.17	—	—	—
SP10	113.8	118.9	45.09	249.8	4.332	30.91
RP10	114.4	119.2	45.13	246.9	5.221	37.28
SP20	114.3	107.3	45.78	249.9	8.253	29.45
RP20	113.6	106.6	45.48	246.4	10.22	36.50
SP30	113.7	93.6	45.66	249.6	11.85	28.21
RP30	113.9	89.9	43.83	249.2	14.58	34.71
SP40	114.5	79.4	45.14	250.1	14.91	26.63
RP40	113.8	78.4	44.62	249.5	18.63	33.27
SP50	113.5	65.0	44.38	250.2	17.63	25.19
RP50	113.7	64.3	43.90	250.2	22.76	32.51
SP60	112.9	50.9	43.46	249.7	21.09	25.11
RP60	113.0	50.3	42.93	248.8	26.84	31.95
PET-fiber	—	—	—	251.5	57.71	41.22

## Data Availability

The data presented in this study are available on request from the corresponding author.
